# Clinical Efficacy and Safety of Bevacizumab, Apatinib, and Recombinant Human Endothelial Inhibitor in the Treatment of Advanced Gastric Cancer

**DOI:** 10.1155/2022/6189833

**Published:** 2022-02-24

**Authors:** Liang Wang, Wei Li, Ya-Gang Liu, Cui Zhang, Wei-Na Gao, Li-Fei Gao

**Affiliations:** ^1^The Second Department of General Surgery, Cangzhou Central Hospital, Cangzhou 061000, Hebei, China; ^2^The Fourth Department of Endocrinology, Cangzhou Central Hospital, Cangzhou 061000, Hebei, China; ^3^The Third Department of General Surgery, Cangzhou Central Hospital, Cangzhou 061000, Hebei, China

## Abstract

**Objective:**

To investigate the clinical efficacy and safety of bevacizumab, apatinib, and recombinant human endothelial inhibitor in the treatment of advanced gastric cancer.

**Methods:**

The medical data of 204 patients with a medium to advanced gastric cancer assessed for eligibility treated in our hospital from February 2019 to April 2020 were retrospectively analyzed. The eligible patients were assigned at a ratio of 1 : 1:1 : 1 to either the control group (chemotherapy), study group I (bevacizumab combined with chemotherapy), study group II (apatinib combined with chemotherapy), or study group III (recombinant human endothelial inhibitor combined with chemotherapy) according to different treatment methods. The treatment efficacy, drug toxicity, quality of life, and serum tumor marker levels before and after treatment were compared among the four groups.

**Results:**

Regarding the treatment effects, the effective rate of study group II (68.63%) was significantly higher than that of the control group (33.33%), study group I (58.82%), and study group III (49.02%) (*P* < 0.05). The four groups showed similar safety and tolerability profiles (*P* > 0.05). The treatment in study group II led to a significantly higher physiological function score vs. the other three groups, but the scores of other items were not significantly different. Significant reduction was observed in the serum tumor markers after treatment in the four groups (*P* < 0.05), but treatment in study group II led to a significantly greater reduction than the other three groups (*P* < 0.05).

**Conclusion:**

The addition of apatinib, bevacizumab, and recombinant human endothelial inhibitor injection to chemotherapy for the treatment of medium to advanced gastric cancer can significantly improve the clinical treatment efficacy, among which the use of apatinib combined with chemotherapy achieves the best results, which is worthy of clinical promotion.

## 1. Introduction

Gastric cancer is a common gastrointestinal malignancy, with its prevalence and mortality rate ranking first among all malignancies in China. Its early clinical manifestations are insidious and nonspecific, which result in the progression of most cases to the advanced stage at the time of diagnosis, at which patients are usually inoperable [1]. For patients with advanced gastric cancer, disease control can only be achieved by chemotherapy. However, most patients are poorly tolerated under chemotherapy, experiencing toxic side effects, unfavorable therapeutic efficacy, and a high risk of deterioration [2]. Moreover, the survival benefits of patients with gastric cancer are modest, with the survival of advanced gastric cancer of about 4 months in the past progressing to 10 months to date. Remarkable efficiency has been achieved by targeted therapy in disease control of tumors. Bevacizumab is a new molecularly targeted antitumor drug in clinical practice, with promising antitumor effects as evidenced by current research reports and data [3–5]. Apatinib is a targeted drug that inhibits the multiplication of venous vascular endothelial cells to suppress the migration of endothelial cells [6–8]. The new recombinant human vascular endothelial inhibitor (Endostar) is a multitarget vascular endothelial inhibitor developed independently in China, which has obtained significant efficacy in the treatment of lung cancer. However, few scholars have been able to draw any systematic comparative research on the efficacy of these three drugs. Accordingly, this study included 204 patients with medium and advanced gastric cancer treated in our hospital from February 2019 to April 2020 as research subjects to evaluate the clinical efficacy and safety of bevacizumab, apatinib, and recombinant human endothelial inhibitor in treating advanced gastric cancer.

## 2. Materials and Methods

### 2.1. General Data

204 patients with a medium to advanced gastric cancer treated in our hospital from February 2019 to April 2020 were identified as research subjects and were assigned to either control group (chemotherapy), study group I (bevacizumab combined with chemotherapy), study group II (apatinib combined with chemotherapy), or study group III (recombinant human endothelial inhibitor combined with chemotherapy) according to different treatment methods, with 51 cases in each group. This study was approved by the ethics committee of our hospital. The inclusion criteria for all study subjects were as follows: patients who were diagnosed with gastric cancer confirmed by pathological and cytological tests, patients aged between 30 and 80 years, and patients with an expected survival of more than 3 months. Exclusion criteria: patients with serious injurious diseases of the heart, liver, kidney, and other important organs; patients with psychiatric diseases; and patients in lactation or pregnancy [9].

### 2.2. Methods

#### 2.2.1. Control Group

Oxaliplatin combined with 5-fluorouracil chemotherapy regimen: on day 1, 90 mg/m^2^ of oxaliplatin (Hangzhou Zhongmei Huadong Pharmaceutical Co., Ltd., National Drug Administration H20113457, specification: 50 mg) was administered by intravenous drip within 3 hours. On days 1–5, 500 mg/m^2^ of 5-fluorouracil (Tianjin Jinyao Amino Acid Co., Ltd., National Drug Administration H12020959, specification: 250 mg) was administered intravenously after the drip. One cycle of chemotherapy spanned 21 days.

#### 2.2.2. Study Group I

Bevacizumab was administered on the basis of the treatment in the control group. Bevacizumab (Roche, Switzerland) was administered at a dose of 7.5 mg/kg by intravenous infusion on day 1, with a cycle of 21 days. The first administration of bevacizumab was performed 2 hours after chemotherapy, and the infusion was monitored for adverse reactions. The following administration of bevacizumab was conducted after chemotherapy. The efficacy of bevacizumab in combination with chemotherapy was evaluated once every cycle, and the treatment was continued in patients with effective cancer control until substantial remission of disease, intolerability, withdrawal of consent, or death.

#### 2.2.3. Study Group II

Apatinib mesylate tablets were administered on the basis of the treatment in the control group. Apatinib mesylate tablets (Jiangsu Hengrui Pharmaceutical Co., Ltd., State Drug Administration H20140103, specification: 0.25 g/tablet) were administered once daily half an hour after meals at an initial dose of 850 mg. Adverse reactions were monitored closely during treatment, and the treatment regimen was adjusted accordingly to allow for treatment tolerance, with a minimum adjusted dose of not less than 500 mg once a day. The treatment was continued until documented disease progression or the development of intolerable adverse effects.

#### 2.2.4. Study Group III

Recombinant human vascular endothelial inhibitor (Endostar) was administered on the basis of the treatment in the control group. Recombinant human vascular endothelial inhibitor (Endostar) (Shandong Xiansheng Medetzin Biopharmaceutical Co., Ltd., State Drug Administration S20050088, specification: 3 mL : 15 mg each) was diluted with 0.9% sodium chloride injection at a volume ratio of 1 : 30 and administered by intravenous drip for 3-4 hours, with an interval of 7 days after 14 days of administration.

The four groups of patients were treated for two cycles of treatment with one course of 21 days. After 42 days of treatment, a comprehensive examination was performed in all four groups. During chemotherapy, patients were closely monitored for bleeding and changes in liver and kidney function and white blood cell counts.

### 2.3. Observation Indexes and Efficacy Criteria

- Efficacy evaluation criteria: if the lesion disappears for more than 4 weeks after treatment, the efficacy is considered markedly effective. If the product of the maximum tumor diameter decreases by half or more after treatment and is maintained for more than 4 weeks, the efficacy is considered effective. If the product of the maximum tumor diameter decreases by less than 50% or increases by less than 25% after treatment, the efficacy is considered ineffective. If new lesions appear after treatment or the product of the maximum tumor diameter increases by more than 25%, the efficacy is considered deteriorated. Total efficacy = (markedly effective + effective)/cases^*∗*^100%.
- The incidence of toxic and side effects was monitored in four groups of patients. According to the National Cancer Institute Common Terminology Criteria for Adverse Events v3.0 (NCI-CTCAEv3.0) [10], the adverse reactions in this study were classified as grades I–IV. The incidence of adverse reactions in patients was also counted, which mainly included thrombocytopenia, cardiovascular toxicity, leukopenia, hemoglobin reduction, abnormal liver and kidney function, and nausea and vomiting.
- The MOS 36-item short-form health survey (SF-36) [11] was used for quality of life measurement. The score of each item was counted and calculated into standard scores, standard scores = (original score-lowest possible score)/the difference value of the highest score and the lowest score × 100%; the sum of the standard scores of each dimension is the total score of SF-36, and higher score indicates better health or functional status.
- The levels of glycoantigen 199 (CA199) and serum carcinoembryonic antigen (CEA) were determined in all groups before and after treatment using the electrochemiluminescence immunoassay [11].

### 2.4. Statistical Analyses

SPSS 26.0 was applied for statistical analysis. The count data were expressed as rates and analyzed using the chi-square test. The measurement data were expressed as mean ± standard deviation ($\bar{x}$ ± *s*), with the *t*-test for the comparison of the means between two groups and one-way ANOVA for the comparison of the means between multiple groups. The differences were statistically significant at *P* < 0.05.

## 3. Results

### 3.1. Comparison of General Data of Four Groups of Patients

There was no significant difference in the general data of the four groups of patients (*P* > 0.05, Table 1).

### 3.2. Comparison of Treatment Efficacy

The total efficacy of patients was 33.33% in the control group, 58.82% in the study I group, 68.63% in the study II group, and 49.02% in the study III group (*P* < 0.05) (Table 2).

### 3.3. Comparison of the Incidence of Adverse Reactions

No statistically significant differences were found in the incidence of thrombocytopenia, cardiovascular toxicity, leukopenia, and abnormal liver and kidney functions among the four groups (*P* > 0.05). The four groups had no serious adverse reactions (Table 3).

### 3.4. Comparison of Quality of Life Scores

The study groups outperformed the control group in terms of quality of life (*P* < 0.05) (Table 4).

### 3.5. Comparison of Serum Tumor Marker Levels

The four groups showed no significant differences in the serum tumor marker levels before treatment (*P* > 0.05). After treatment, the levels of CEA and CA199 were significantly decreased in the four groups (*P* < 0.05), in which the three study groups obtained significantly lower results than the control group (*P* < 0.05), and the study group II showed remarkably lower outcomes than the study groups I and III (*P* < 0.05) (Table 5).

## 4. Discussion

Gastric cancer is a common malignancy with insidious and variable symptoms in the early stage. The disease has mostly progressed to the advanced stage by the time of diagnosis. Patients with advanced gastric cancer are mostly inoperable, with great difficulty in treatment, poor prognosis, and high mortality rates. Statistically, more than 50% of patients undergoing surgery are associated with in situ recurrence or distant metastasis, which undermines patients' physical and mental health and compromises their quality of life. Currently, chemotherapy is the mainstay for treating advanced gastric cancer, which underscores the significance of the scientific application of chemotherapy drugs to enhance the treatment efficiency and avoid adverse reactions. In tumor growth and metastasis, neovascularization is a major influencing factor, so the exploration of therapeutic methods targeting antitumor angiogenesis remains a key issue to be addressed. Previous studies have revealed that angiogenesis inhibitors could coordinate or even superimpose the effects of cytotoxic agents or radiation therapy [5, 6].

Bevacizumab is a monoclonal antibody that inhibits the vascular endothelial growth factor and is mainly used for metastatic cancer treatment, with the mechanism to inhibit the mitosis of tumor vascular endothelial cells and angiogenesis by blocking or reducing the binding of the vascular endothelial growth factor to its corresponding receptor. In addition, it can lead to insufficient blood oxygenation of growing and multiplying tumor cells by disrupting tumor vasculature to facilitate the contact of chemotherapeutic drugs with tumors [12, 13]. Apatinib is a derivative compound of PTK787 and is the first generation of small-molecule tyrosine kinase inhibitor targeting the angiogenic signaling pathway developed clinically in China and the latest oral targeted therapeutic agent against tumor angiogenesis. It effectively binds ligands such as vascular endothelial growth factor VEG-FR-2 and blocks the signaling after VEGF [14] binding, thereby inhibiting tumor neovascularization [15]. Recombinant human vascular endothelial inhibitor is an endogenous factor that inhibits neovascularization by binding and phagocytosing proteins of endothelial cells to block their signaling pathways and to mediate the expression profile of endothelial cells. The efficacy of recombinant human vascular endothelial inhibitor on tumors is mediocre during single-use but can be potentiated in a joint administration. Cisplatin features an extremely strong peritoneal nodal penetration, and docetaxel and tegafur capsules can disrupt the microvascular meshwork, which, together with the inhibitory and modulatory effects of recombinant human endothelial inhibitor, effectively boosts the therapeutic effect. Prior studies have confirmed the significant clinical efficacy of bevacizumab, apatinib, and recombinant human endothelial inhibitor in combination with chemotherapy in the treatment of patients with a medium to advanced gastric cancer [16, 17].

In this study, the higher efficacy in all three study groups versus the control group indicated that the combination of chemotherapy with apatinib, bevacizumab, and recombinant human endothelial inhibitor potentiated the effectiveness of chemotherapy drugs in inhibiting tumor growth. In terms of treatment efficiency, the study group II outperformed the study group I, followed by the study group III, indicating that the efficacy of apatinib combined with chemotherapy was better than that of bevacizumab and recombinant human endothelial inhibitor combined with chemotherapy. Assumedly, apatinib highly selectively competes with intracellular vascular endothelial growth factor receptor 2, and binds to it, thereby inhibiting tumor angiogenesis and reducing tumor blood supply, oxygen supply, and other nutrients supply to tumor growth [17–19]. The absence of statistically significant differences in the incidence of adverse reactions among the four groups indicates the treatment regimens used in the study groups did not significantly increase the toxicity of the drugs with a high safety profile [20, 21]. The three study groups presented superior improvement in the quality of life to that of the control group; additionally, the levels of CEA and CA199 were markedly reduced in the four groups after treatment, with better results in the study groups, indicating that the combined treatment regimens employed in this study yielded more favorable outcomes than the stand-alone chemotherapy. Furthermore, the study group II showed remarkably lower levels of CEA and CA199 versus the study groups I and III.

## 5. Conclusion

The addition of apatinib, bevacizumab, and recombinant human endothelial inhibitor injection to chemotherapy for the treatment of medium to advanced gastric cancer can significantly improve the clinical treatment efficacy, among which the use of apatinib combined with chemotherapy achieves the best results, which is worthy of clinical promotion.

## Figures and Tables

**Table 1:** Comparison of general data of the four groups of patients (‾*x* ± *s*).

Groups	Gender (male/female) (case)	Mean age (year)	Mean course of disease (months)	Stage (case)			
				IIIa	IIIb	IIIc	IV
Control group	35/16	48.62 ± 6.87	4.61 ± 1.53	11	20	11	9
Study I group	32/19	51.04 ± 7.98	5.02 ± 2.31	10	22	10	9
Study II group	36/15	50.04 ± 5.34	4.87 ± 2.75	11	21	9	10
Study III group	34/17	49.77 ± 8.23	4.91 ± 1.83	12	19	10	10
*X* ^2^/*F*	0.482	0.975	0.331	0.232	0.408	0.249	0.129
*P*	0.546	0.406	0.803	0.975	0.939	0.969	0.988

**Table 2:** Comparison of treatment efficacy of patients in the four groups (*n* (%)).

Groups	*n*	Markedly effective	Effective	Ineffective	Deteriorated	Total efficacy
Control group	51	4	13	27	7	33.33%
Study group I	51	8	22	18	2	58.82%^a^
Study group II	51	10	25	15	1	68.63%^a^
Study group III	51	6	19	23	3	49.02%^a^
*X* ^2^						15.259
*P*						0.002

^a^Statistically significant difference in comparison with the control group.

**Table 3:** Comparison of the incidence of adverse reactions among the four groups of patients (*n* (%)).

Groups	*n*	Thrombocytopenia		Cardiovascular toxicity		Leukopenia		Hemoglobin reduction		Abnormal liver and kidney functions		Nausea and vomiting	
		I-II	III-IV	I-II	III-IV	I-II	III-IV	I-II	III-IV	I-II	III-IV	I-II	III-IV
Control group	51	20	17	15	14	19	14	18	10	18	12	22	11
Study group I	51	21	15	15	13	21	17	15	11	19	17	25	13
Study group II	51	20	14	14	11	17	17	14	13	18	16	23	12
Study group III	51	23	16	16	16	19	18	17	16	20	16	26	14

**Table 4:** Comparison of quality of life scores among patients in the four groups (‾*x* ± *s*, points).

Groups	Physical functioning	Bodily pain	Social functioning	Role emotional	Mental health	Vitality	General health
Control group	69.31 ± 5.76	54.41 ± 4.35	49.87 ± 4.12	50.11 ± 5.64	61.87 ± 6.85	59.32 ± 7.13	65.97 ± 4.63
Study group I	79.11 ± 5.48	55.28 ± 5.79	53.46 ± 6.75	51.34 ± 4.97	70.55 ± 5.49	61.55 ± 6.82	78.11 ± 6.31
Study group II	83.57 ± 6.49	56.34 ± 5.91	54.51 ± 5.74	54.73 ± 5.17	74.66 ± 6.53	66.33 ± 7.28	78.94 ± 7.03
Study group III	80.42 ± 6.34	55.30 ± 3.36	53.36 ± 4.93	52.44 ± 4.23	74.32 ± 6.78	65.09 ± 4.87	77.57 ± 4.82
*F*							61.95
*P*							0.279

**Table 5:** Comparison of serum tumor marker levels before and after treatment in the four groups (‾*x* ± *s*, mg/L).

Groups	*n*	CEA		CA199	
		Before treatment	After treatment	Before treatment	After treatment
Control group	51	67.51 ± 13.48	33.14 ± 5.53^*∗*^	436.75 ± 52.64	246.34 ± 30.01^*∗*^
Study group I	51	68.31 ± 12.37	18.47 ± 5.34^*∗*^^a^	431.79 ± 48.99	185.37 ± 26.51^*∗*^^a^
Study group II	51	68.49 ± 11.64	10.36 ± 6.13^*∗*^^a^	435.17 ± 49.61	163.74 ± 27.16^*∗*^^a^
Study group III	51	67.99 ± 12.08	17.97 ± 5.83^*∗*^^a^	434.64 ± 50.36	190.34 ± 29.44^*∗*^^a^
*F*		88.233	141.582	73.213	78.839
*P*		0.021	<0.001	0.011	<0.001

^*∗*^ *P* < 0.05, in the comparison with pretreatment. ^a^*P* < 0.05, in the comparison between the study groups and the control group after treatment.

## Data Availability

The datasets used to support the findings of this study are available from the corresponding author upon request.
